# Telerehabilitation for Stroke Survivors: Systematic Review and Meta-Analysis

**DOI:** 10.2196/10867

**Published:** 2018-10-26

**Authors:** Huidi Tchero, Maturin Tabue Teguo, Annie Lannuzel, Emmanuel Rusch

**Affiliations:** 1 Unit of Wounds and Healing Department of Trauma and Orthopaedic Surgery Centre Hospitalier Louis Constant Fleming Saint Martin Saint Martin, Guadeloupe France; 2 Centre Hospitalier Universitaire de Guadeloupe Université des Antilles Guadeloupe France; 3 Le Centre de Recherche Inserm - U1219 Université de Bordeaux Bordeaux France; 4 Institut de santé publique, d'épidémiologie et de développement Université de Bordeaux Bordeaux France; 5 Le Gérontopôle du Centre Hospitalier Universitaire de Toulouse Toulouse France; 6 Department of Neurology University Hospital Center Pointe-à-Pitre Guadeloupe France; 7 Service d'Information Médicale, Epidémiologie et Economie de la Santé Centre Hospitalier Régional Universitaire de Tours Tours France

**Keywords:** meta-analysis, mobile phone, rehabilitation, stroke, telemedicine

## Abstract

**Background:**

Telerehabilitation is an emerging technology through which medical rehabilitation care can be provided from a distance.

**Objective:**

This systematic review and meta-analysis aims to investigate the efficacy of telerehabilitation in poststroke patients.

**Methods:**

Eligible randomized controlled trials (RCTs) were identified by searching MEDLINE, Cochrane Central, and Web of Science databases. Continuous data were extracted for relevant outcomes and analyzed using the RevMan software as the standardized mean difference (SMD) and 95% CI in a fixed-effect meta-analysis model.

**Results:**

We included 15 studies (1339 patients) in our systematic review, while only 12 were included in the pooled analysis. The combined effect estimate showed no significant differences between the telerehabilitation and control groups in terms of the Barthel Index (SMD –0.05, 95% CI –0.18 to 0.08), Berg Balance Scale (SMD –0.04, 95% CI –0.34 to 0.26), Fugl-Meyer Upper Extremity (SMD 0.50, 95% CI –0.09 to 1.09), and Stroke Impact Scale (mobility subscale; SMD 0.18, 95% CI –0.13 to 0.48]) scores. Moreover, the majority of included studies showed that both groups were comparable in terms of health-related quality of life (of stroke survivors), Caregiver Strain Index, and patients’ satisfaction with care. One study showed that the cost of telerehabilitation was lower than usual care by US $867.

**Conclusions:**

Telerehabilitation can be a suitable alternative to usual rehabilitation care in poststroke patients, especially in remote or underserved areas. Larger studies are needed to evaluate the health-related quality of life and cost-effectiveness with the ongoing improvements in telerehabilitation networks.

## Introduction

Telemedicine is the exchange of medical information from one location to another using electronic communication to achieve clinical health care from a distance [1]. These technologies allow communication between medical staff and patients, as well as the transmission of imaging and other health information data from one place to another [2]. It can be used to accelerate medical emergency services in conditions with narrow therapeutic windows, such as stroke [3] and myocardial infarction [4], and facilitate access to medical services that would not often be available in rural communities [2].

Stroke rehabilitation therapy aims to improve the patients’ motor function, health-related quality of life (HRQoL), and psychological well-being [5]. Successful rehabilitation depends on stroke severity, rehabilitation team skills, and the cooperation of patients and their families [6]. However, many patients have reduced access to care because of limited regional and logistic resources; these patient groups could benefit from a system that allows a health professional to provide rehabilitation services from a remote location [7]. A physical medicine or rehabilitation specialist at a hospital can observe patients as they execute movements and monitor their improvement. Quantitative data, such as range of motion and physical force, can be recorded and transported through the network to the hospital for review [8].

Over the past decade, a number of randomized controlled trials (RCTs) have investigated the benefits of telerehabilitation in poststroke patients in comparison to usual rehabilitation methods. These studies showed that telerehabilitation was either equal [9,10] or superior [11,12] to usual rehabilitation in terms of improvements in the activities of daily living and psychological status of patients and their caregivers. This study aims to investigate the benefits of telemedicine in poststroke rehabilitation in a meta-analysis framework.

## Methods

This study was performed and reported following the Preferred Reporting Items for Systematic Reviews and Meta-Analyses checklist for systematic reviews of intervention (Multimedia Appendix 1).

### Literature Search and Study Selection

On January 14, 2018, we performed a comprehensive search of the following databases: MEDLINE (via PubMed), Cochrane Central, and Web of Science. The following keywords were used with different combinations: “Telemedicine,” “Telestroke,” “Telerehabilitation,” “Stroke,” and “Brain infarction” with no filters applied (either by language or period of publication). Multimedia Appendix 2 provides the search strategies of the 3 used databases. In addition, we searched the clinical trial register “Clinicaltrials.gov” for any unpublished or ongoing studies. Furthermore, a manual screening of the bibliography of included studies was performed for any studies we missed during the electronic search.

Studies were considered eligible for inclusion if they fulfilled the following criteria: (1) assessed the efficacy of different telerehabilitation models in poststroke patients and (2) employed an RCT design. We excluded studies that had a nonrandomized or single-arm design or that examined the technical components of the telerehabilitation systems. Two reviewers screened the search results using the criteria mentioned above in 2 subsequent steps: title and abstract screening, followed by full-text screening. When the judgments of both reviewers were not similar, another reviewer solved the discrepancy.

### Data Extraction and Outcomes

Two independent reviewers used a preformatted Excel sheet to extract data for the prespecified outcomes, including (1) activities of daily living: Barthel Index and Berg Balance Scale; (2) motor function: Action Research Arm Test (ARAT), Fugl-Meyer Upper Extremity (FM-UE), and Stroke Impact Scale (mobility subscale); (3) HRQoL outcomes and satisfaction with care; and (4) cost-effectiveness. Data were extracted as the mean (SD) of change before and after treatment and then was compared between groups. When these values were not given in the included studies, they were calculated using the equations in the Cochrane Handbook for Systematic reviews of intervention [13]. When numerical data were not available for these outcomes or could not be reliably extracted, they were analyzed in a qualitative approach.

During the extraction, we also evaluated the risk of different forms of bias in the included studies. We used the Cochrane risk of bias tool [13], which deals with the following sources of bias: (1) selection bias (random sequence generation and allocation concealment); (2) performance bias (blinding of participants and outcome assessors); (3) attrition bias (incomplete outcome data); (4) reporting bias (selective reporting); and (5) other sources of bias.

### Statistical Analysis and Outcome Interpretation

We used the RevMan software (Version 5.3; Cochrane Collaboration, Oxford, England) to perform all statistical analyses in this study. Based on the nature of extracted data (continuous), they were pooled as the standardized mean difference (SMD) with 95% CI, using the inverse variance meta-analysis method. *P*<.05 was considered significant for the effect estimate. The analysis was done first under the fixed-effect model for assuming homogeneity; in case of heterogeneity, we shifted to the random-effects model. Heterogeneity was assessed using the chi-square test (*P*<.10 was considered significant for between-study heterogeneity), and its extent was measured using the *I*^2^ test.

## Results

### Results of Literature Search

**Figure 1:**
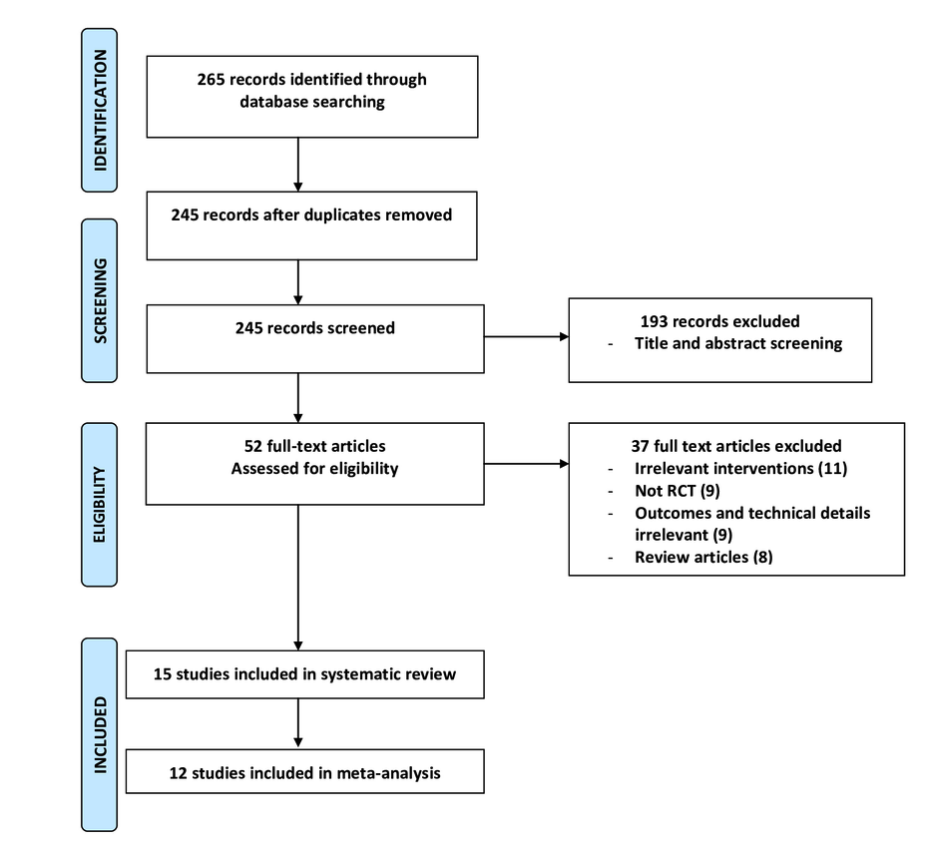
The Preferred Reporting Items for Systematic Reviews and Meta-Analyses (PRISMA) flow diagram of the study selection process. RCT: randomized controlled trial.

Our literature search retrieved 245 unique records, which were reduced to 52 records after the title and abstract screening. After a meticulous full-text screening, 15 studies (1339 patients) were identified as eligible for our systematic review [9-12,14-24], while only 12 [9-11,16-24] were included in our meta-analysis (1246 patients). Figure 1 shows the details of our screening process.

### Characteristics of Included Studies

The included RCTs had a sample size ranging between 9 and 536 patients. They compared different models of telerehabilitation to standard rehabilitation care or a home-based exercise program. The follow-up period in these studies ranged between 4 and 24 weeks. Multimedia Appendix 3 summarizes the design of included studies, components of the used telerehabilitation systems, and the baseline characteristics of enrolled patients.

### Risk of Bias Assessment Results

**Figure 2:**
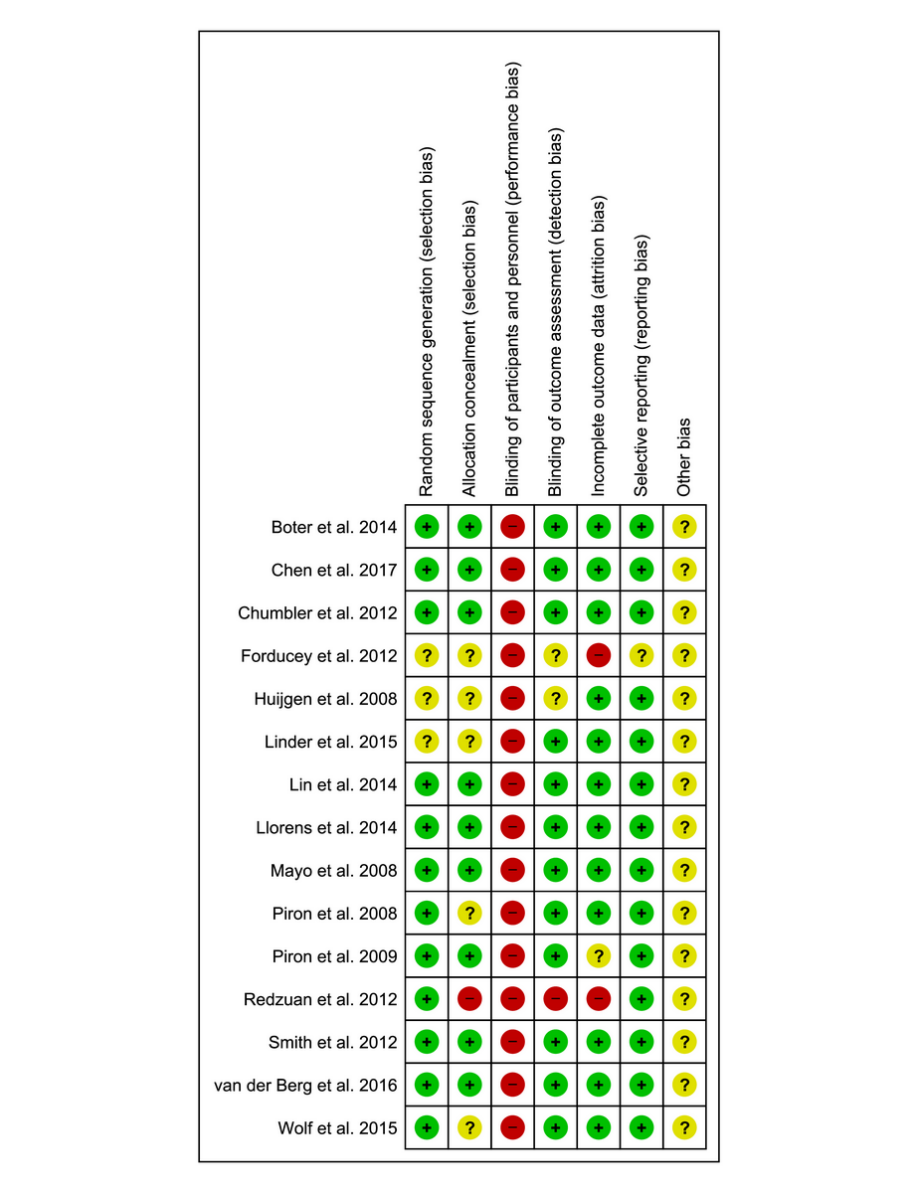
Risk of bias assessment summary according to the Cochrane risk of bias tool: Red, green, and yellow colours indicates high, low, and unclear risk of bias, respectively.

All included studies reported adequately on their methods of random sequence generation, blinding of outcome assessors, and reducing the risk of attrition bias, except for 3 trials in each domain. Owing to the nature of the intervention, blinding participants was not possible in all included trials. Only 9 studies reported adequately on their methods of allocation concealment [9-12,14,17,19-21]. Figure 2 summarizes the risk of bias assessment results, with red, green, and yellow colors indicating high, low, and unclear risk of bias, respectively.

### Results of Outcome Assessment

#### Activities of Daily Living and Balance Function

##### Barthel Index

**Figure 3:**
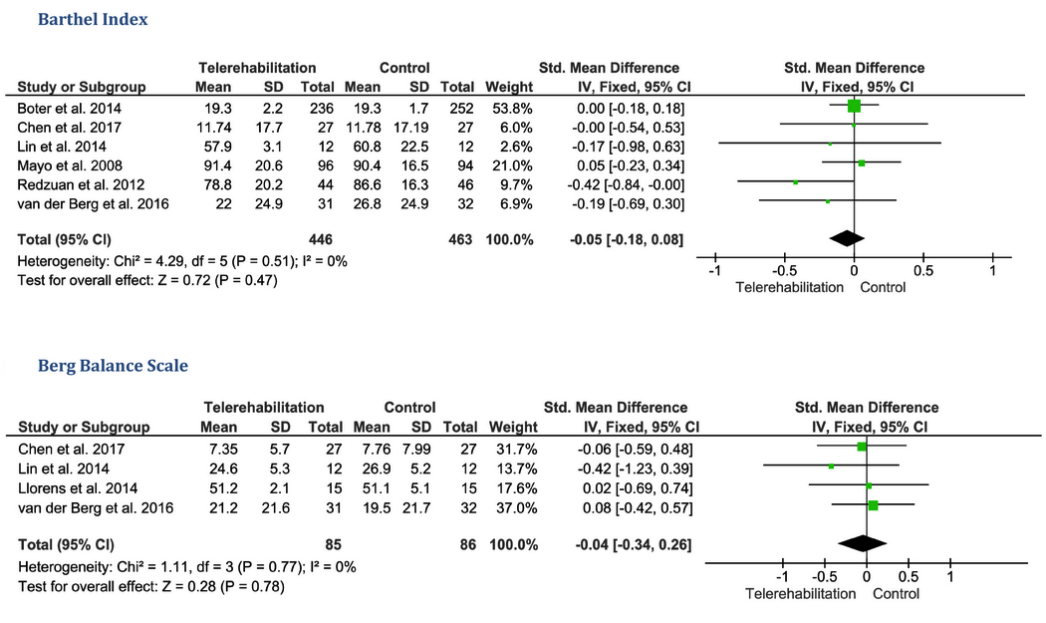
The pooled standardized mean difference between the telerehabilitation and control groups in terms of Barthel Index and Berg Balance Scale scores.

Under the fixed-effect model, pooling data from 6 trials [9-11,17,20,23] showed no significant difference between the telerehabilitation and control groups in terms of the Barthel Index score (SMD –0.05, 95% CI –0.18 to 0.08, *P*=.47, 909 patients). Pooled studies were homogenous (*P*=.51, *I*^2^=0%; Figure 3).

##### Berg Balance Scale

Under the fixed-effect model, the pooled analysis of data from 4 studies [10,11,17,19] showed no significant difference between the telerehabilitation and control groups in terms of the Berg Balance Scale (SMD –0.04, 95% CI –0.34 to 0.26, *P*=.78, 171 patients). Pooled studies were homogenous (*P*=.77, *I*^2^=0%; Figure 3).

#### Motor Function

In this study, different scales were used to assess this outcome.

##### Fugl-Meyer Upper Extremity

**Figure 4:**
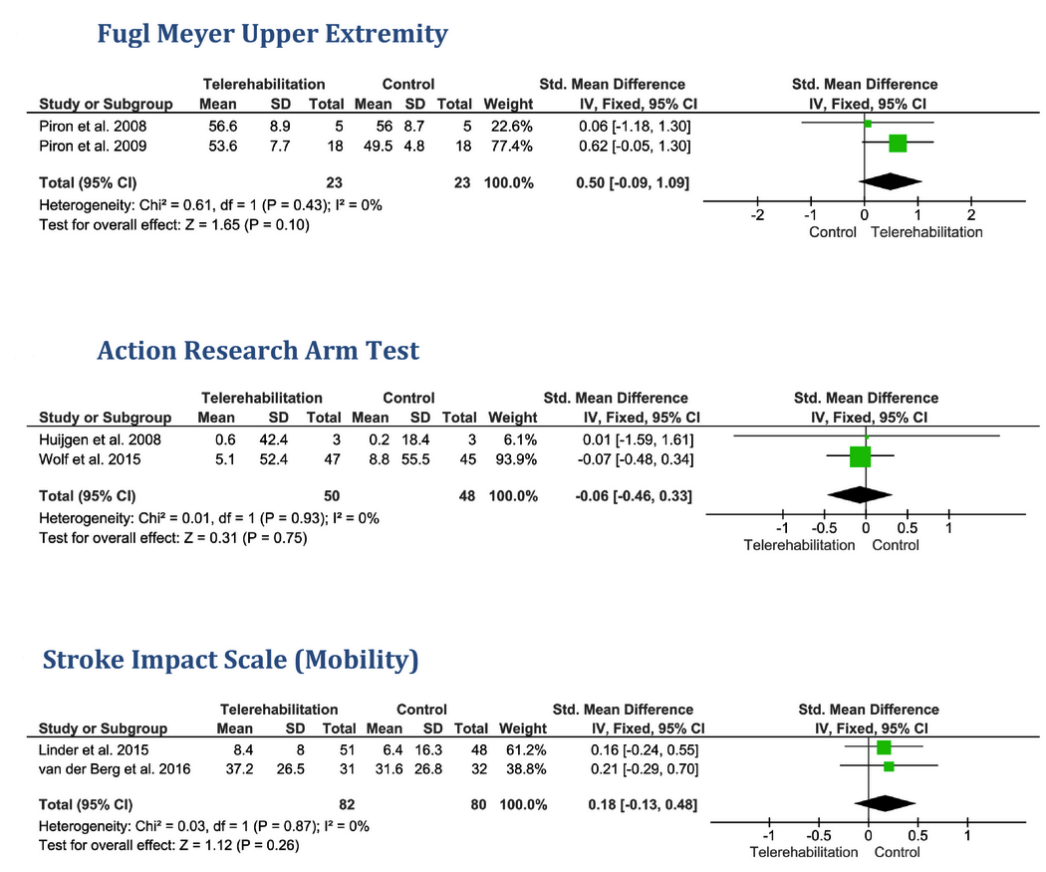
The pooled standardized mean difference between the telerehabilitation and control groups in terms of Fugl-Meyer Upper Extremity, Action Research Arm Test, and Stroke Impact Scale (mobility) scores.

Two homogenous studies (*P*=.43, *I*^2^=0%) reported data on the mean FM-UE score in both groups [21,22]. Under the fixed-effect model, the pooled effect estimate showed no significant difference (SMD 0.50, 95% CI –0.09 to 1.09, *P*=.10, 46 patients) between the telerehabilitation and control groups with regard to FM-UE (Figure 4).

##### Action Research Arm Test

Two homogenous studies (*P*=.93, *I*^2^=0%) provided data on the mean ARAT score in both groups [16,24]; therefore, the analysis was conducted under the fixed-effect model. No significant difference was noted between both groups in terms of the ARAT score between the telerehabilitation and control groups (SMD –0.06, 95% CI –0.46 to 0.33, *P*=.75, 98 patients; Figure 4).

##### Stroke Impact Scale—Mobility Subscale

Under the fixed-effect model, the pooled effect estimate of 2 studies [11,18] showed no significant difference (SMD 0.18, 95% CI –0.13 to 0.48, *P*=.26, 162 patients) between the telerehabilitation and control groups in terms of the Stroke Impact Scale-mobility subscale score. Pooled studies were homogenous (*P*=.87, *I*^2^=0%; Figure 4).

#### Patients’ Quality of Life

Six studies reported on the HRQoL of poststroke patients. Boter reported that telerehabilitation patients achieved better scores on the Short-Form (SF-36) emotional role limitation (mean difference=7.9, 95% CI 0.1 to 15.7) than the control group [9]. However, Forducey et al and Mayo et al showed no significant differences (*P*>.05) between both groups with regard to the SF-12 and the physical component score of the SF-36, respectively [15,20].

Two studies used 2 different version of the Functional Independence Measure—self-administered and telephone versions. Both studies recorded no differences between the telerehabilitation and control groups [14,15]. Interestingly, Smith et al compared the effects of both treatments on the outcomes of mastery, self-esteem, and social support and reported no significant effects of either treatment on these outcomes [12].

In addition, 4 of 6 studies assessed the treatment effects on depression. Two studies by Linder et al and Smith et al recorded no differences between both rehabilitation methods (telerehabilitation and usual rehabilitation) on the Center for Epidemiological Studies-Depression scale in poststroke patients [12,18]. Similarly, Boter et al and Mayo et al reported no significant difference between both groups on the Hospital Depression Scale and the Geriatric Depression Scale [9,20]. Only one study by Redzuan et al reported comparable rates of poststroke complications in these groups but did not specify the nature of these complications [23].

#### Caregivers’ Quality of Life

Five included studies reported on the quality of life in caregivers of poststroke patients. Of these, 4 studies showed no significant difference (*P*<.05) between the 2 rehabilitation modalities (telerehabilitation and usual rehabilitation) in terms of the Caregiver Strain Index [9-11,23]. On the other hand, Smith et al reported that caregivers in the telerehabilitation group had lower depression scores than those in the usual care group [12].

#### Satisfaction With Care

Three studies reported on the patient satisfaction with care in both groups. Of these, 2 these studies showed no significant difference between the telerehabilitation and usual care groups in terms of patient satisfaction scores [9,17]. While Piron et al randomized 10 patients into a virtual reality virtual reality-telerehabilitation group and virtual reality hospital-based intervention. Using a modified satisfaction questionnaire, they reported that tele-virtual reality patients achieved equal or higher scores to hospital-based virtual realityR patients in almost all points; however, a significant difference in motor performance was only noted in the tele-virtual reality group [22].

#### Cost-Effectiveness

Only one study of high methodological quality (according to the Cochrane risk of bias tool) by Lloréns et al reported data on the cost-effectiveness outcome. They calculated that for 1 participant, the cost of telerehabilitation was lower than that of usual care by about US $654 (US $1490.23 and US $853.61 for in-clinic and home rehabilitation programs, respectively) [19]. Although setting the virtual reality system at home required US $800, the telerehabilitation arm required fewer work hours by physical therapists and it eliminated the cost of round trips to the clinic with every session.

## Discussion

This systematic review showed that patients on telerehabilitation achieve comparable restoration of daily of life activities and HRQoL to those on usual care rehabilitation. Moreover, caregivers of stroke survivors in both groups had a comparable quality of life (as assessed by the Caregiver Strain Index), and one study [12] reported lower rates of depression in the telerehabilitation group. Satisfaction with care remains a problem in poststroke rehabilitation as most included studies showed that telerehabilitation failed to improve the patients’ satisfaction with care.

The comparable improvement in motor performance in the telerehabilitation and usual care groups was evident on all motor assessment scales; this adds to the reliability of our findings that telerehabilitation can produce significant motor improvements. Moreover, a previous meta-analysis showed that when telemedicine is used to deliver thrombolytic therapy for stroke patients, it produced comparable rates of favorable clinical outcomes at 90 days as those produced by in-hospital thrombolysis [3]. Therefore, telemedicine can assist with improving the motor function from the onset of stroke, not only during rehabilitation. The improved motor performance would further translate into improved activities of daily living and HRQoL.

Another interesting finding of our review is that telerehabilitation produced comparable improvement of the patients’ HRQoL to usual rehabilitation; this extended beyond stroke survivors to include their caregivers as well. This is essential because several studies have shown high rates of depression and quality of life impairment among caregivers of stroke survivors, which negatively influences their supportive functions [25,26]. However, research on the caregiver’s quality of life and interventions to improve their performance is not adequate. Therefore, confirming the value of telemedicine in this regard should be a focus of future studies.

Compared with usual rehabilitation, telerehabilitation offers several advantages, including easier access, mentoring for disabled stroke patients, and the ability of patients to self-record on their pain, mood, and activity [27]. Unfortunately, several barriers limit the spreading of telerehabilitation; these barriers include administrative licensing, medicolegal ambiguity, and financial sustainability [28]. Another barrier, especially in low-income countries (where telerehabilitation would be most needed), is the lack of technological infrastructure. A cross-sectional study (on 100 stroke survivors) in a Ghanaian outpatient neurology clinic demonstrated that 80%-93% of patients had a positive attitude toward telerehabilitation interventions; however, only 35% of them had smartphones [29]. Further development of telerehabilitation networks is essential to overcome these barriers [30].

The included studies used different models of telerehabilitation. For example, some studies used only telephone calls, while others used videoconferencing, educational videos, Web-based chats, and virtual reality systems (as illustrated in Multimedia Appendix 3). Moreover, the duration of rehabilitation programs and frequency of follow-up visits or contact with medical staff differed from a study to another. So far, there are no adequate data in the literature about which model or telerehabilitation tool is optimal for these patients and thus future head-to-head comparative studies are advised.

Regarding the cost-effectiveness, the included study by Llorens et al showed lower cost (by US $654) for the telerehabilitation program than the in-clinic program with similar efficacy. This is in agreement with several previous studies in other patient populations that showed that telerehabilitation could reduce the cost of disease management and patient rehabilitation. The reduction in cost is mostly due to avoiding travel (especially from remote areas where telemedicine is most useful) [31,32]. However, the evidence is conflicting on this outcome, and further studies are needed to confirm it [33,34].

Our systematic review has some strengths. We performed a comprehensive literature search and reported our methodology according to the Preferred Reporting Items for Systematic Reviews and Meta-Analyses checklist and the Cochrane Handbook for Systematic Reviews of Interventions. Compared with a former systematic review of 10 studies by Laver et al [35], we investigated the effects of telemedicine on several outcomes—activities of daily living, motor performance, HRQoL, satisfaction with care, and cost-effectiveness. Moreover, we evaluated the benefits of telemedicine not only on stroke survivors but also on the caregiver’s quality of life.

However, our meta-analysis has some limitations. First, the relatively small sample size in included studies restricts the generalizability of our findings. Second, some outcomes could not be analyzed quantitatively because of the heterogeneity of data in the included studies (different scales of measurements or data formats). Moreover, we could not assess the risk of publication bias because according to Egger et al, funnel plot-based methods are not accurate for <10 included studies per outcome [36].

Hence, larger RCTs are required to confirm the current evidence and provide more data on outcomes such as HRQoL and cost-effectiveness. Data reporting should be performed in a clear standardized format to enable reliable extraction for future meta-analysis studies. Our search of clinicaltrials.gov registry retrieved 19 ongoing studies that are active or still recruiting participants such as NCT02665052, NCT02360488, and NCT01157195. The results of these studies are eagerly awaited. Moreover, it would be interesting for clinicians to investigate the benefits of using telerehabilitation to supplement the usual care. Of note, these trials should not necessarily show that telerehabilitation achieves high outcomes, but confirmation of comparable outcomes is needed.

In conclusion, telerehabilitation can be a suitable alternative to usual rehabilitation care in poststroke patients. This may have potential implications for patients, especially in remote or underserved areas. Nevertheless, larger studies are needed to evaluate the quality of life and cost-effectiveness with the ongoing advances in telerehabilitation systems.

## Appendix

Multimedia Appendix 1 Preferred Reporting Items for Systematic Review and Meta-Analyses (PRISMA) checklist.

Multimedia Appendix 2 Literature search strategy.

Multimedia Appendix 3 Risk of bias assessment in included randomized controlled trials.

## Abbreviations

ARAT: Action Research Arm Test
FM-UE: Fugl-Meyer Upper Extremity
HRQoL: health-related quality of life
SF: short-form
SMD: standardized mean difference
RCT: randomized controlled trial

## References

[1] Smith AC, Bensink M, Armfield N, Stillman J, Caffery L (2005). Telemedicine and rural health care applications. J Postgrad Med.

[2] Matusitz J, Breen Gerald-Mark (2007). Telemedicine: its effects on health communication. Health Commun.

[3] Baratloo A, Rahimpour L, Abushouk A, Safari S, Lee C, Abdalvand A (2018). Effects of Telestroke on Thrombolysis Times and Outcomes: A Meta-analysis. Prehosp Emerg Care.

[4] de Waure Chiara, Cadeddu C, Gualano M, Ricciardi W (2012). Telemedicine for the reduction of myocardial infarction mortality: a systematic review and a meta-analysis of published studies. Telemed J E Health.

[5] Hz N, Brady B, Mcgahan L, Teasell R, Skidmore B, Tj D (2003). Canadian Coordinating Office for Health Technology Assessment.

[6] de Bustos EM, Vuillier F, Chavot D, Moulin T (2009). Telemedicine in stroke: organizing a network–rationale and baseline principles. Cerebrovasc Dis.

[7] Holden M, Dyar T, Dayan-Cimadoro Lilian (2007). Telerehabilitation using a virtual environment improves upper extremity function in patients with stroke. IEEE Trans Neural Syst Rehabil Eng.

[8] Cikajlo I, Rudolf M, Goljar N, Burger H, Matjačić Zlatko (2012). Telerehabilitation using virtual reality task can improve balance in patients with stroke. Disabil Rehabil.

[9] Boter H, HESTIA Study Group (2004). Multicenter randomized controlled trial of an outreach nursing support program for recently discharged stroke patients. Stroke.

[10] Chen J, Jin W, Dong WS, Jin Y, Qiao FL, Zhou YF, Ren Cheng Chuan (2017). Effects of Home-based Telesupervising Rehabilitation on Physical Function for Stroke Survivors with Hemiplegia: A Randomized Controlled Trial. Am J Phys Med Rehabil.

[11] van den Berg Maayken, Crotty M, Liu E, Killington M, Kwakkel G, van Wegen E (2016). Early Supported Discharge by Caregiver-Mediated Exercises and e-Health Support After Stroke: A Proof-of-Concept Trial. Stroke.

[12] Smith G, Egbert N, Dellman-Jenkins M, Nanna K, Palmieri P (2012). Reducing depression in stroke survivors and their informal caregivers: a randomized clinical trial of a Web-based intervention. Rehabil Psychol.

[13] Higgins J, Green S (2008). Cochrane Handbook for Systematic Reviews of interventions. Cochrane Handbook for Systematic Reviews of Interventions.

[14] Chumbler N, Quigley P, Li X, Morey M, Rose D, Sanford J (2012). Effects of telerehabilitation on physical function and disability for stroke patients: a randomized, controlled trial. Stroke.

[15] Forducey PG, Glueckauf RL, Bergquist TF, Maheu MM, Yutsis M (2012). Telehealth for persons with severe functional disabilities and their caregivers: facilitating self-care management in the home setting. Psychol Serv.

[16] Huijgen B, Vollenbroek-Hutten M, Zampolini M, Opisso E, Bernabeu M, Van NJ (2008). Feasibility of a home-based telerehabilitation system compared to usual care: arm/hand function in patients with stroke, traumatic brain injury and multiple sclerosis. Journal of telemedicine and telecare.

[17] Lin K, Chen C, Chen Y, Huang W, Lai J, Yu S (2014). Bidirectional and multi-user telerehabilitation system: clinical effect on balance, functional activity, and satisfaction in patients with chronic stroke living in long-term care facilities. Sensors.

[18] Linder S, Rosenfeldt A, Bay R, Sahu K, Wolf S, Alberts J (2015). Improving Quality of Life and Depression After Stroke Through Telerehabilitation. Am J Occup Ther.

[19] Lloréns Roberto, Noé Enrique, Colomer C, Alcañiz Mariano (2015). Effectiveness, usability, and cost-benefit of a virtual reality-based telerehabilitation program for balance recovery after stroke: a randomized controlled trial. Arch Phys Med Rehabil.

[20] Mayo N, Nadeau L, Ahmed S, White C, Grad R, Huang A (2007). Bridging the gap: the effectiveness of teaming a stroke coordinator with patient's personal physician on the outcome of stroke. Age and ageing.

[21] Piron L, Turolla A, Agostini M, Zucconi C, Cortese F, Zampolini M (2009). Exercises for paretic upper limb after stroke: a combined virtual-reality and telemedicine approach. Journal of Rehabilitation Medicine.

[22] Piron L, Turolla A, Tonin P, Piccione F, Lain L, Dam M (2008). Satisfaction with care in post-stroke patients undergoing a telerehabilitation programme at home. Journal of telemedicine and telecare.

[23] Redzuan N, Engkasan J, Mazlan M, Abdullah S (2012). Effectiveness of a video-based therapy program at home after acute stroke: a randomized controlled trial. Archives of physical medicine and rehabilitation.

[24] Wolf SL, Sahu K, Bay RC, Buchanan S, Reiss A, Linder S (2015). The HAAPI (Home Arm Assistance Progression Initiative) trial: a novel robotics delivery approach in stroke rehabilitation. Neurorehabilitation and neural repair.

[25] Cimarolli VR, Reinhardt JP, Horowitz A (2006). Perceived overprotection: support gone bad. J Gerontol B Psychol Sci Soc Sci.

[26] Visser-Meily A, Post M, van de Port I, Maas C, Forstberg-Wärleby G, Lindeman E (2009). Psychosocial functioning of spouses of patients with stroke from initial inpatient rehabilitation to 3 years poststroke: course and relations with coping strategies. Stroke.

[27] Hjelm N (2005). Benefits and drawbacks of telemedicine. Journal of telemedicine and telecare.

[28] Akbik F, Hirsch J, Chandra RV, Frei D, Patel A, Rabinov J (2017). Telestroke-the promise and the challenge. Part two-expansion and horizons. Journal of neurointerventional surgery.

[29] Sarfo F, Adamu S, Awuah D, Sarfo-Kantanka O, Ovbiagele B (2017). Potential role of tele-rehabilitation to address barriers to implementation of physical therapy among West African stroke survivors: A cross-sectional survey. Journal of the neurological sciences.

[30] Donoso BEV, McCoy S, Fechko A, Price R, Gilbertson T, Moritz C (2014). Preliminary investigation of an electromyography-controlled video game as a homeprogram for persons in the chronic phase of stroke recovery. Archives of physical medicine and rehabilitation.

[31] Fauchier L, Sadoul N, Kouakam C, Briand F, Chauvin M, Babuty D, Clementy J (2005). Potential cost savings by telemedicine-assisted long-term care of implantable cardioverter defibrillator recipients. Pacing Clin Electrophysiol.

[32] Thaker DA, Monypenny R, Olver I, Sabesan S (2013). Cost savings from a telemedicine model of care in northern Queensland, Australia. Med J Aust.

[33] Zhai Y, Zhu W, Cai Y, Sun D, Zhao J (2014). Clinical- and cost-effectiveness of telemedicine in type 2 diabetes mellitus: a systematic review and meta-analysis. Medicine (Baltimore).

[34] Whitten PS, Mair FS, Haycox A, May CR, Williams TL, Hellmich S (2002). Systematic review of cost effectiveness studies of telemedicine interventions. BMJ.

[35] Laver K, Schoene D, Crotty M, George S, Lannin N, Sherrington C (2013). Telerehabilitation services for stroke. Cochrane Database Syst Rev.

[36] Egger M, Davey SG, Schneider M, Minder C (1997). Bias in meta-analysis detected by a simple, graphical test. BMJ.

