# Supplementary material for: Telerehabilitation for Stroke Survivors: Systematic Review and Meta-Analysis
Source: J Med Internet Res. 2018 Oct 26;20(10):e10867. doi: 10.2196/10867 (PMC6250558; doi:10.2196/10867)
Supplement: Multimedia Appendix 2 [file jmir_v20i10e10867_app2.pdf]

Supplementary File 2: Literature Search Strategy

The exact used keywords for all searched databases were:

1. Stroke
2. Ischemic Stroke
3. Cerebral Infarction
4. Cerebrovascular Accident
5. Poststroke
6. Stroke Survivors
7. Telemedicine
8. Telestroke
9. Telerehabilitation
10. Rehabilitation
11. Home Intervention
12. Telesupervising Rehabilitation
13. Outreach Care
14. Videoconferencing

The generally used search strategy was 1 OR 2 OR 3 OR 4 OR 5 OR 6 OR 7 AND 8 OR 9 OR 10 OR 11 OR 12 OR 13 OR 14. No restrictions by language or date were applied allover.

Special attributes

- PubMed: The Mesh database was searched for both main words (Stroke and Telerehabilitation) and results were added from the Mesh database to the search builder. The search results were initially large; therefore, we used the Tile and Abstract filter: Results = 128.

- Cochrane Central: We searched in the Title, Abstract and Keywords (Using the Trials Filter in the Cochrane Library): Results = 42

- Web of Science: We used the Advanced Search interface on the Web of Science Core Collection: Results = 86
