# Supplementary material for: Telerehabilitation for Stroke Survivors: Systematic Review and Meta-Analysis
Source: J Med Internet Res. 2018 Oct 26;20(10):e10867. doi: 10.2196/10867 (PMC6250558; doi:10.2196/10867)
Supplement: Multimedia Appendix 3 [file jmir_v20i10e10867_app3.pdf]

Reason(s) for author judgement	Risk of Bias	Supplementary File 3: Risk of bias assessment in included randomized controlled trialsXBoter et al. 2014
A minimization procedure was performed to reduce imbalance in the distributions of treatment numbers within the levels of each individual possible prognostic factor.	Low	Random sequence generation
Not reported but unlikely to have happened given the nature of the intervention	High	Blinding of patients
The assessment by telephone was performed in a blinded fashion.	Low	Blinding of outcome assessors
Allocation was done by means of a central telephone service	Low	Concealment of allocation
The percentage of patient withdrawal was less than 10% and an intention to treat analysis was performed	Low	Incomplete data reporting
All assigned outcomes were adequately reported	Low	Selective outcome reporting
	Unclear	Others
**Reason(s) for author judgement**	**Risk of Bias**	**Chen et al. 2017**
A blocked randomization sequence was computer generated	Low	Random sequence generation
In this study, given the nature of the intervention, it was impossible to blind the survivors, the caregivers, and the therapists about allocation and intervention	High	Blinding of patients
Only outcome assessors and statisticians were blinded	Low	Blinding of outcome assessors
Allocation concealment was ensured, as allocation information was protected in opaque sealed enevelopes	Low	Concealment of allocation
The percentage of patient withdrawal was less than 10% and an intention to treat analysis was performed	Low	Incomplete data reporting
All assigned outcomes were adequately reported	Low	Selective outcome reporting
	Unclear	Others
**Reason(s) for author judgement**	**Risk of Bias**	**Chumbler et al. 2012**
Eligible study participants were randomized by centrally sealed allocation into the STeleR or usual care (UC) groups.	Low	Random sequence generation
The study was a 3-site, 2-arm, single-blinded RCT	High	Blinding of patients
Through telephone interview by a research assistant blinded to randomization, and through the medical record.	Low	Blinding of outcome assessors
Eligible study participants were randomized by centrally sealed allocation into the STeleR or usual care (UC) groups.	Low	Concealment of allocation
Intention-to-treat analyses were used for all outcomes.	Low	Incomplete data reporting
All assigned outcomes were adequately reported	Low	Selective outcome reporting
	Unclear	Others
**Reason(s) for author judgement**	**Risk of Bias**	**Forducey et al. 2012**
Following screening and informed consent, each subject was randomly assigned into either the intervention (videophone technology) or the control (standard home care)	Unclear	Random sequence generation
Not reported but unlikely to have happened given the nature of the intervention	High	Blinding of patients
Not reported	Unclear	Blinding of outcome assessors

Not reported	Unclear	Concealment of allocation
Two patients withdrawed and not reported if have been included in an intenton to treat analysis	High	Incomplete data reporting
	Unclear	Selective outcome reporting
	Unclear	Others
**Reason(s) for author judgement**	**Risk of Bias**	**Huijgen et al. 2008**
Each time HCAD was available, three patients were randomly assigned: two to the intervention group and the other one to the control group. The randomization was performed at each individual clinical centre.	Unclear	Random sequence generation
Not reported but unlikely to have happened given the nature of the intervention	High	Blinding of patients
Not reported	Unclear	Blinding of outcome assessors
Not reported	Unclear	Concealment of allocation
For all analyses an intention-to-treat analysis, including patients with protocol deviations was performed.	Low	Incomplete data reporting
All assigned outcomes were adequately reported	Low	Selective outcome reporting
	Unclear	Others
**Reason(s) for author judgement**	**Risk of Bias**	**Lin et al. 2014**
The randomization was performed by random numbers which were generated by computer.	Low	Random sequence generation
Not reported but unlikely to have happened given the nature of the intervention	High	Blinding of patients
One physical therapist performed the pre- and post-assessments for both groups and blinded to the assignment.	Low	Blinding of outcome assessors
The rater was blinded to the allocation of participants.	Low	Concealment of allocation
Analyses of intention to treat were used for one drop-out.	Low	Incomplete data reporting
All assigned outcomes were adequately reported	Low	Selective outcome reporting
	Unclear	Others
**Reason(s) for author judgement**	**Risk of Bias**	**Linder et al. 2015**
The method of randomization is not adequately reported	Unclear	Random sequence generation
A prospective, multisite, single-blind, randomized controlled clinical trial was designed	High	Blinding of patients
All evaluations were completed by a physical therapist or occupational therapist blinded to group assignment at baseline and end of treatment (EOT).	Low	Blinding of outcome assessors
Not reported	Unclear	Concealment of allocation
For the purposes of this intent-to-treat analysis, data were assumed to be missing at random.	Low	Incomplete data reporting
All assigned outcomes were adequately reported	Low	Selective outcome reporting
	Unclear	Others
**Reason(s) for author judgement**	**Risk of Bias**	**Llorens et al. 2014**
Randomization was computer-generated using a basic random number generator in a ratio of 1:1.	Low	Random sequence generation
Single-blind randomized controlled trial.	High	Blinding of patients
A physical therapist (PTA), blind to the intervention, was responsible for assessing the participants and for supervising and adjusting their	Low	Blinding of outcome assessors

training.		
The allocation sequence was concealed from an independent researcher. A sealed envelope identifying the group of each participant was given to the therapists to inform them of the allocation.	Low	Concealment of allocation
Only one patient dopped out from the study	Low	Incomplete data reporting
All assigned outcomes were adequately reported	Low	Selective outcome reporting
	Unclear	Others
**Reason(s) for author judgement**	**Risk of Bias**	**Mayo et al. 2008**
Randomization was done at discharge, in random blocks of four, six or eight	Low	Random sequence generation
A stratified, balanced, evaluator-blinded, randomized clinical trial was carried out	High	Blinding of patients
A stratified, balanced, evaluator-blinded, randomized clinical trial was carried out	Low	Blinding of outcome assessors
Sealed evelopes were prepared in advance	Low	Concealment of allocation
with an 'intention-to-treat' approach using linear regression	Low	Incomplete data reporting
All assigned outcomes were adequately reported	Low	Selective outcome reporting
	Unclear	Others
**Reason(s) for author judgement**	**Risk of Bias**	**Piron et al. 2008**
Using simple randomization, the subjects were assigned to two different groups of five patients.	Low	Random sequence generation
Not reported but unlikely to have happened given the nature of the intervention	High	Blinding of patients
The examining physician was blind to the type of treatment given and evaluated arm motor performance in all patients, both before and after therapy	Low	Blinding of outcome assessors
Not adequately reported	Unclear	Concealment of allocation
No patients withdrawed from the study	Low	Incomplete data reporting
All assigned outcomes were adequately reported	Low	Selective outcome reporting
	Unclear	Others
**Reason(s) for author judgement**	**Risk of Bias**	**Piron et al. 2009**
Selected patients were assigned to 2 groups according to a simple randomization technique using sequentially numbered, opaque sealed envelopes	Low	Random sequence generation
Randomized single-blind controlled trial.	High	Blinding of patients
The examining neurologist was blind to the treatments administered to the patients.	Low	Blinding of outcome assessors
Selected patients were assigned to 2 groups according to a simple randomization technique using sequentially numbered, opaque sealed envelopes	Low	Concealment of allocation
Data on patient withdrawal or intention to treat analysis are not reported	Unclear	Incomplete data reporting
All assigned outcomes were adequately reported	Low	Selective outcome reporting
	Unclear	Others
**Reason(s) for author judgement**	**Risk of Bias**	**Redzuan et al. 2012**
A blocked randomization sequence (block of 10) was generated using a computer program	Low	Random sequence generation
Given the nature of the intervention, it was not possible to blind	High	Blinding of patients

participants and caregivers from knowing what group they were in.		
The randomization list was kept by 1 of the investigators who was involved in patient recruitment and assessment.	High	Blinding of outcome assessors
The randomization list was kept by 1 of the investigators who was involved in patient recruitment and assessment.	High	Concealment of allocation
Sixteen patients withdrawed and were not included in the analysis	High	Incomplete data reporting
All assigned outcomes were adequately reported	Low	Selective outcome reporting
	Unclear	Others
**Reason(s) for author judgement**	**Risk of Bias**	**Smith et al. 2012**
Randomization was conducted via computer by PP.	Low	Random sequence generation
Not reported but unlikely to have happened given the nature of the intervention	High	Blinding of patients
Assessors were blind to condition	Low	Blinding of outcome assessors
Allocation involved a permuted block design with blocks of random length so that the final sample included 16 dyads per condition.	Low	Concealment of allocation
Data were thus analyzed separately in terms of both intent to treat	Low	Incomplete data reporting
All assigned outcomes were adequately reported	Low	Selective outcome reporting
	Unclear	Others
**Reason(s) for author judgement**	**Risk of Bias**	**van der Berg et al. 2016**
A statistician external to the study generated the random sequence in random blocks of 2 to 6 using a computer software program	Low	Random sequence generation
Participants and treating physiotherapists could not be masked to intervention group allocation.	High	Blinding of patients
By an independent assessor blinded to allocation.	Low	Blinding of outcome assessors
Created sequentially numbered, sealed opaque envelopes containing group allocation for participants.	Low	Concealment of allocation
Data were analyzed according to the intention-to-treat principle, with the statistician masked to group allocation.	Low	Incomplete data reporting
Outcome measures have been detailed and fully referenced in a previously published protocol paper	Low	Selective outcome reporting
	Unclear	Others
**Reason(s) for author judgement**	**Risk of Bias**	**Wolf et al. 2015**
An adaptive, stratified, computer-driven randomization procedure was used for group assignment to balance critical participant characteristics	Low	Random sequence generation
The protocol and design for this prospective, multisite, single-blind, randomized controlled clinical trial have been described in our previous publication	High	Blinding of patients
Participants were assessed before randomization (T1) and after completion of the intervention (T2) by occupational/ physical therapists who were trained in the use of standardized assessment protocols and blinded to participant group assignment.	Low	Blinding of outcome assessors
Not reported	Unclear	Concealment of allocation
For purposes of this intent-to-treat analysis, we assumed that data were missing at random	Low	Incomplete data reporting
All assigned outcomes were adequately reported	Low	Selective outcome reporting
	Unclear	Others
